# Mortality in association with antipsychotic medication use and clinical outcomes among geriatric psychiatry outpatients with COVID-19

**DOI:** 10.1371/journal.pone.0258916

**Published:** 2021-10-21

**Authors:** Bienvenida Austria, Rehana Haque, Sukriti Mittal, Jamie Scott, Aninditha Vengassery, Daniel Maltz, Wentian Li, Blaine Greenwald, Yun Freudenberg-Hua

**Affiliations:** 1 Division of Geriatric Psychiatry, Zucker Hillside Hospital, Glen Oaks, NY, United States of America; 2 Donald and Barbara Zucker School of Medicine at Hofstra/Northwell, Hempstead, NY, United States of America; 3 Information Services, Product Services and Management, Northwell Health, Lake Success, NY, United States of America; 4 Center for Genomics and Human Genetics, The Feinstein Institutes for Medical Research, Manhasset, NY, United States of America; 5 Litwin-Zucker Center for Alzheimer’s Disease, The Feinstein Institutes for Medical Research, Manhasset, NY, United States of America; University of South Carolina College of Pharmacy, UNITED STATES

## Abstract

**Objectives:**

Older adults are particularly vulnerable to the negative consequences of antipsychotic exposure and are disproportionally affected by higher mortality from coronavirus disease 2019 (COVID-19). Our goal was to determine whether concurrent antipsychotic medication use was associated with increased COVID-19 mortality in older patients with preexisting behavioral health problems. We also report on findings from post-COVID follow-ups.

**Design:**

Retrospective observational study.

**Participants:**

Outpatients at a geriatric psychiatric clinic in New York City.

**Measurements:**

Demographic and clinical data including medication, diagnosis and Clinical Global Impression Severity (CGI-S) scales on outpatients who had COVID-19 between February 28^th^ and October 1^st^ 2020 were extracted from the electronic health records (EHR) from the hospital.

**Results:**

A total of 56 patients were diagnosed with COVID-19 (mean age 76 years; median age 75 years) and 13 (23.2%) died. We found an increased mortality risk for patients who were prescribed at least one antipsychotic medication at the time of COVID-19 infection (Fisher’s exact test P = 0.009, OR = 11.1, 95% confidence interval: 1.4–96.0). This result remains significant after adjusting for age, gender, housing context and dementia (Logistic regression P = 0.035, Beta = 2.4). Furthermore, we found that most patients who survived COVID-19 (88.4%) recovered to pre-COVID baseline in terms of psychiatric symptoms. Comparison of pre- and post-COVID assessments of CGI-S for 33 patients who recovered from COVID-19 were not significantly different.

**Conclusion:**

We observed a higher COVID-19 mortality associated with concurrent antipsychotics use in older patients receiving behavioral health services. The majority of patients in our geriatric clinic who recovered from COVID-19 appeared to return to their pre-COVID psychiatric function. More precise estimates of the risk associated with antipsychotic treatment in older patients with COVID-19 and other underlying factors will come from larger datasets and meta-analyses.

## Introduction

Long-term antipsychotic medication use has been unequivocally associated with increased mortality risk in older adults [1–3]. Among many adverse medical effects, antipsychotics have been reported to be associated with an increased risk for serious respiratory adverse events [4, 5] with a link to potential immune dysfunction [6]. The Coronavirus disease (COVID-19) caused by the SARS-CoV-2 virus infection disproportionally affects elderly with higher morbidity and mortality [7, 8]. The infection fatality rate was estimated to reach 4.9% for people aged 65–74 years and 14.2% for those 75 years and older [7]. In addition to older age, more risk factors have been identified, including but not limited to gender [9, 10], dementia, living at institutional care facilities, and medical comorbidities [11, 12]. Some of these risk factors may not be independent; for example, people with dementia are also more likely to live in care facilities. A population based observational study showed that living in care facilities is associated with an increased risk of COVID-19 mortality in comparison with living in independent housing, potentially through exposure to visitors and care workers, and due to poor underlying health [13].

Given that older adults are particularly vulnerable to the negative consequence of antipsychotic exposure as well as to COVID-19, we evaluated clinical data from a community-based geriatric psychiatric outpatient clinic in New York City (NYC) to determine whether long-term use of antipsychotics in the context of COVID-19 would increase the risk of mortality. Moreover, neurological and psychiatric complications are common during the acute phase of COVID-19, including delirium [14], stroke, intracerebral hemorrhage, CNS vasculitis, encephalopathy, new-onset psychosis, neurocognitive syndrome and affective symptoms [15, 16]. An increased incidence of psychiatric disorders has been reported in early post-COVID follow-ups [17, 18] and psychiatric patients may require additional mental health support during the pandemic [18, 19]. Therefore, we wonder whether older patients with preexisting mental illness show more post-COVID psychiatric consequences. We took advantage of our out-patient follow-ups to compare pre- and post COVID-19 overall clinical psychiatric symptom severity as a secondary outcome.

## Methods

This study is a single-center retrospective observational study and approved by the Institutional Review Board of Northwell Health (# 20–0200). The outpatients and their caregivers reported COVID-19 diagnosis and related symptoms to the treating psychiatrists at the Zucker Hillside Hospital (ZHH) of the Northwell Health system. We included all patients who had COVID-19 between February 28^th^ and October 1^st^ 2020. The COVID-19 diagnosis was confirmed with reverse transcription polymerase chain reaction (RT-PCR) during the symptomatic phase or confirmed using serology test after the suspected acute COVID-19 infection. The outcome is defined as whether the patient expired during the acute COVID-19 hospitalization. Clinical data including the use of antipsychotics at the time of COVID-19 (exposure variable), antidepressants, age, gender, dementia diagnosis (defined as ICD-10 codes starting with F01, F02, F03, F04, F09, G30, G31, and G32) as well as Clinical Global Impression severity scales (CGI-S) [20] were extracted from hospital EHR and manually quality checked. The CGI-S, which is integrated in the EHR but not as a required field, is routinely used by the treating psychiatrists during clinical assessments. CGI-S scales from September 1^st^ 2019 to the date of COVID-19 for each patient are defined as pre-COVID (T0) and from the date of COVID-19 till October 22^nd^ 2020 (time point of EHR data extraction) are defined as post-COVID (T1) measures. Living arrangement, estimated timing of COVID-19 infection, and whether the patients survived were obtained through chart review. We estimated the date of COVID-19 infection using the date of the reported symptom onset by patients and their caregivers or the date of positive results of the RT-PCR test. In addition to CGI-S, the treating psychiatrists (who typically follow the same patients longitudinally) were surveyed in October 2020 for their impression of whether the patients recovered to their pre-COVID functional baseline. The acute symptoms of COVID-19 were treated at various health facilities including urgent care centers, primary care physicians’ and medical specialists’ offices and inpatient medical facilities. Following the Strengthening the Reporting of Observational Studies in Epidemiology (STROBE) statement [21], a check list is presented as **S1 Table**.

Statistical analysis was performed using R version 3.5.0 [22]. Given the limited sample sizes we use Fisher’s exact test to test for univariate association between binary variables such as patient survival with antipsychotic use [23]. The odds ratio (OR) and 95% confidence interval are calculated using standard methods [24]. We used multivariable logistic regression to account for potential confounding factors including age, gender, living arrangement, and dementia diagnosis. As a sensitivity analysis, antidepressant use was added to the model. Shapiro-Wilk normality test indicated non-normal distribution of differences between CGI-S at T0 and T1 (P = 0.001). Accordingly, we used both paired t-test to compare the means and Wilcoxon paired signed rank test to compare the medians of individual pre- (T0) and post-COVID-19 (T1) CGI-S.

## Results

A total of 56 patients (mean age 76.0 ± 8.5 years; median age: 74.5 years; interquartile range: 13.0 years) (**Table 1; S2 Table**) with confirmed COVID-19 were reported to the treating psychiatrists between February and September 2020. Thirteen (23.2%) patients died while being hospitalized for confirmed COVID-19. In this cohort, gender is not significantly associated with mortality (Fisher’s exact test P = 0.31) but higher age is associated with higher risk of death (P = 0.05; Beta = 0.08), which is consistent with previously reported findings [7]. In our cohort, female patients had a higher mean age than male patients (77.8 vs. 71.5 years old) (t-test P = 0.01). Institutional living arrangement (Fisher’s exact test P = 0.10) and dementia diagnosis (P = 0.16) are not significantly associated with COVID-19 mortality in this cohort.

**Table 1:** Patient characteristics stratified by outcome of COVID-19 patients.

		Survived	Expired	OR (95% CI)	P
N (Total = 56)		43	13		
age_covid (mean (SD))		74.72 (8.04)	80.15 (9.15)	1.1 (1.0–1.2)	0.05
gender (%)	F	29 (67.4)	11 (84.6)	2.6 (0.5–27.5)	0.31
	M	14 (32.6)	2 (15.4)		
Institutional living (%)	Yes	6 (14.0)	5 (38.5)	3.7 (0.7–19.4)	0.10
	No	37 (86.0)	8 (61.5)		
Dementia (%)	Yes	10 (23.3)	6 (46.2)	2.8 (0.6–12.4)	0.16
	No	33 (76.7)	7 (53.8)		
Antipsychtics (%)	Yes	22 (51.2)	12 (92.3)	11.1 (1.4–96.0)	0.009
	No	21 (48.8)	1 (7.7)		

The association between survival and age was tested by logistic regression, whereas to test the associations of binary variables, Fisher’s exact test was used. All P-values are unadjusted for covariables. Institutional Living is defined as living at an assisted living facility or at a rehabilitation facility. Antipsychotics: patients who were prescribed at least one antipsychotic medication at the time of COVID-19 infection to treat preexisting behavioral health disorders.

**Fig 1:**
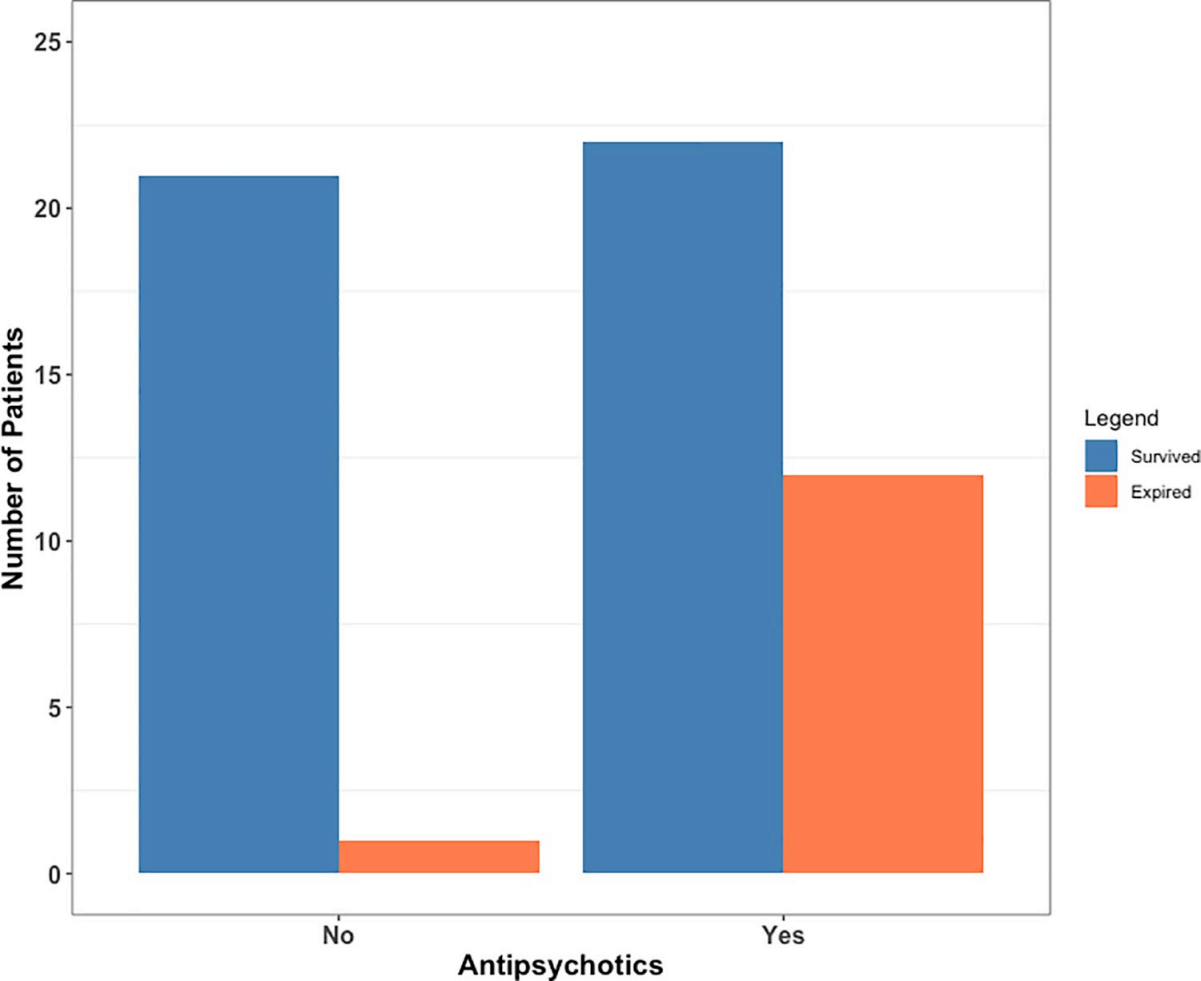
COVID-19 mortality of patients stratified by antipsychotics use. Number of geriatric psychiatric patients who survived and died from COVID-19. X-axis: “No”: not being prescribed an antipsychotic medication (steel blue); “Yes”: being prescribed an antipsychotic medication before COVID-19 (coral).

We observed a significant association between being prescribed an antipsychotic medication at the time of COVID-19 and an increased mortality risk (Fisher’s exact test P = 0.009, OR = 11.1, 95% confidence interval: 1.4–96.0) (**Fig 1**). The association between antipsychotics and COVID-19 mortality remained significant (P = 0.035, Beta = 2.4) after adjusting for age, gender, living arrangements (home vs. institutions), and dementia diagnosis. Further adjusting for antidepressants use as a sensitivity analysis didn’t change the association (**S3 Table**). In univariable analysis, antipsychotics use was not significantly associated with age, gender, living arrangements or dementia (**S4 Table**). Among the 34 patients treated with antipsychotics, only two (5.9%) received monotherapy with a typical antipsychotic medication (haloperidol) and both recovered. Another two patients were prescribed a combination of a typical and an atypical antipsychotic medication and both expired. The remaining 30 patients were all taking atypical antipsychotics and 10 of them (33.3%) expired. The typical antipsychotics used are haloperidol, paliperidone, fluphenazine, trifluoperazine and the atypical antipsychotics include olanzapine, aripiprazole, quetiapine, risperidone, and clozapine. These antipsychotics were prescribed for a variety of conditions including dementia with behavior disorders, bipolar disorder, schizophrenia, schizoaffective disorder, psychosis, and major depressive disorder. Other psychotropic medications include mood stabilizers such as lamotrigine and valproic acid, gabapentin, anxiolytics such as benzodiazepines or buspirone and cholinesterase inhibitors such as donepezil.

**Fig 2:**
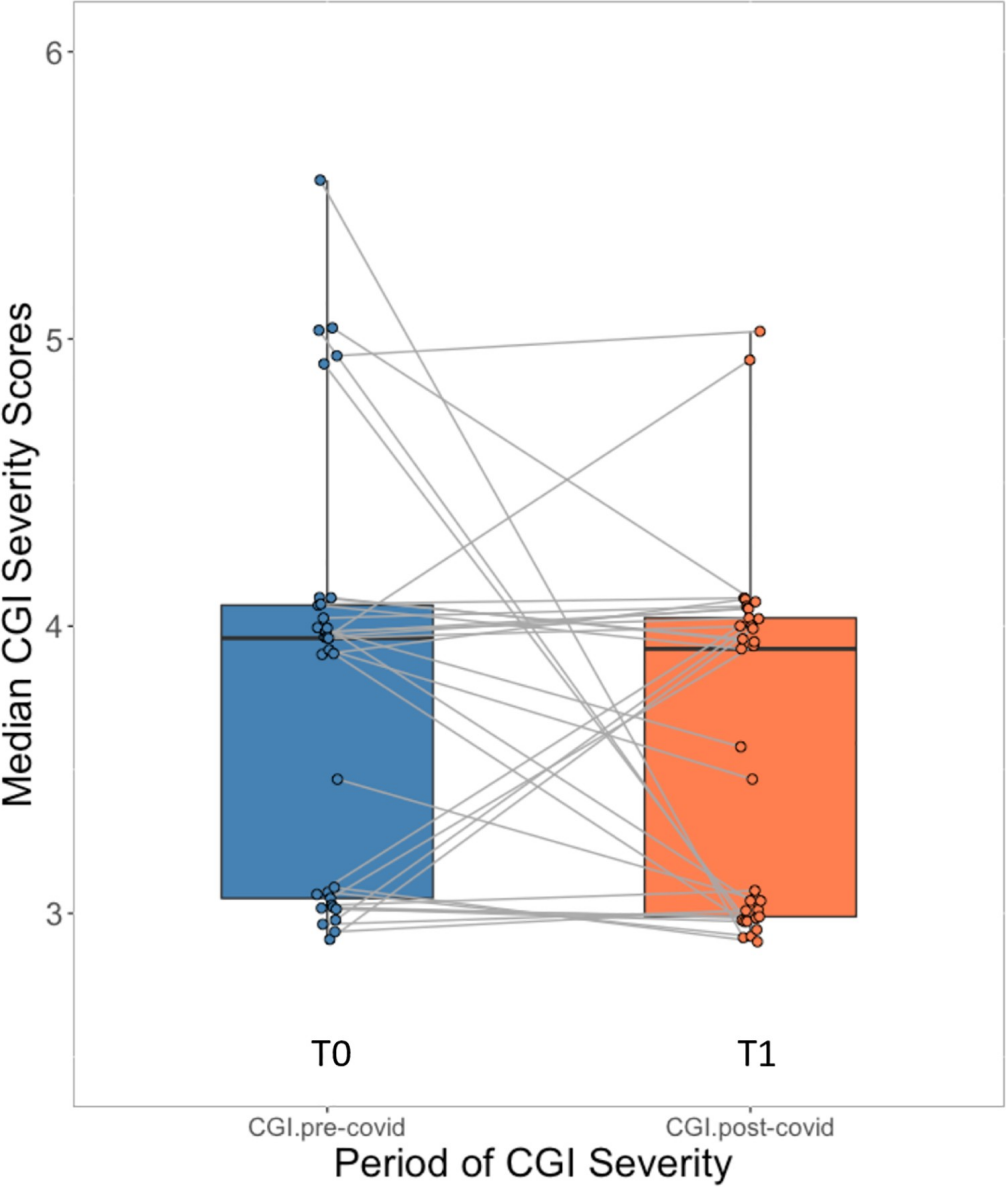
Median CGI-S scores before and after COVID-19. “CGI.pre-covid” (T0): Median Clinical Global Impression severity scales (CGI-S) before COVID-19 (steel blue); “CGI.post-covid” (T1): Median CGI-S during follow-up after COVID-19 (coral). Each point represents the median CGI-S score of one patient and the gray lines connect the pre- and post-COVID CGI-S for each patient. Thirty-three patients have both pre- and post-COVID CGI-S scores (Paired t-test for mean P = 0.23, Wilcoxon paired signed rank test for median: P = 0.35).

The out-patient setting, in which patients being followed up by the same psychiatrist, further presents an opportunity to investigate how elderly patients with behavioral health conditions who survived COVID-19 recovered longitudinally in terms of psychiatric function. When surveyed, the treating geriatric psychiatrists reported that the majority of the patients—38 out of 43 patients (88.4%)—recovered to pre-COVID level of functioning. In addition, 33 patients had at least one CGI-S [20] score—a structured rating on how mentally ill a patient presents—for both pre- and post-COVID visits. Comparing the aggregated T0 and T1 CGI-S scores, we found no significant difference (paired t-test for differences of the mean CGI-S: P = 0.23 and Wilcoxon paired signed rank test for median: P = 0.35) (**Fig 2**).

## Discussion

As older adults are particularly vulnerable to adverse consequence of antipsychotic exposure [1–3] and have higher mortality risk from COVID-19 [7, 8, 25], we evaluated EHR data from a community-based geriatric psychiatry clinic during the height of the COVID-19 pandemic in NYC. We investigated whether concurrent antipsychotics prescription is associated with increased risk of COVID-19 mortality in this geriatric population receiving behavioral health services. We found that concurrent antipsychotic exposure was associated with an increased mortality risk in context of acute COVID-19. This finding remains significant after controlling for age, gender, living arrangement, and the presence of dementia.

A previous report showed that patients with a pre-existing psychiatric diagnosis while hospitalized for COVID-19 had a mortality rate that is twice as high compared to those without a psychiatric condition [26]. The authors speculated that there may be a causal relationship between psychiatric disorders and/or psychotropic medication and the immune system. A systematic screening of protein-protein interactions to identify critical factors for SARS-CoV-2 infection found that typical antipsychotics may have antiviral effects against COVID-19 while atypical antipsychotics do not [27]. As a proof of concept, the authors then utilized medical billing data to confirm their drug repurposing targets and found that half as many of the new users of typical antipsychotics (N = 13) compared with the new users of atypical antipsychotics (N = 26) progressed to the point of requiring mechanical ventilation. Another observational study found a reduced risk for intubation or death from COVID-19 to be associated with antidepressants as home medications, if they were continued within 48 hours of hospital admission [28]. A significant association of antipsychotics use with intubation or death (Hazard ration = 1.98) was observed in the crude analysis, but not in the multivariate analysis of this earlier study. These findings underscore both the importance and complexity of investigating psychotropic medication use and COVID-19 outcomes.

We found that despite mental status changes being a common symptom in COVID-19, geriatric behavioral health patients in our study did not show worsening of psychiatric function during the short-term post-COVID period. There had been considerations that COVID-19 may potentially trigger future neurodegeneration through neuroinflammatory mechanisms [29]. One large study that included adults at all ages indicated a bidirectional interplay between COVID-19 and psychiatric problems [17]. They found that survivors of COVID-19 appear to be at increased risk of psychiatric sequelae including dementia, and a psychiatric diagnosis might be an independent risk factor for negative outcomes of COVID-19. A potential mechanism may include damage to blood-brain barrier mediated by a massive increase in circulating pro-inflammatory factors [30]. It is critical that COVID-19 survivors receive longitudinal follow-up neuropsychiatric assessments to monitor and investigate the long-term neurocognitive outcome.

Our study has several limitations. First, the odds ratio for the association of antipsychotics and mortality had wide confidence interval that is likely due to the small sample size. Second, adjusting for covariables in a small sample may lead to overfitting of the multiple logistic regression model. Third, mortality could be affected by additional demographic and clinical factors [31] that were not accounted for, which further limits the generalizability of our findings. Despite the sample size limitation, our exploratory results nevertheless present an addition to the discussion on the interplay between COVID-19 and mental health [18] as well as the post-COVID recovery [32]. Given the retrospective observational character of our study, we emphasize that although we report a significant correlation between long-term antipsychotic medication usage and COVID-19 mortality, any hypothetical causal relation remains to be understood. Antipsychotics are frequently prescribed in geriatric patients to treat a wide spectrum of disorders. Our study is statistically underpowered to investigate the effects of subtypes of antipsychotic medication as well as the combined effects of different psychotropics and underlying behavioral health conditions.

## Conclusions

We observed a higher COVID-19 mortality associated with antipsychotics use in older patients receiving behavioral health services. The majority of COVID-19 survivors recovered without overt psychiatric sequelae during short-term follow-ups. Future studies with larger data sets and meta-analysis will provide more precise estimates of the degree of risk of antipsychotic treatment in the elderly with comorbid COVID-19 so that providers and consumers can consider risks, benefits, and alternatives.

## Supporting information

S1 Table STROBE checklist. (DOCX)

S2 Table Deidentified characteristics of patients. “0” =“Yes, “1” = No. For the purpose of deidentification, we included age range of the patients. Institutional Living is defined as living at an assisted living facility or at a rehabilitation facility. Antipsychotics: patients who were prescribed at least one antipsychotic medication at the time of COVID-19 infection to treat preexisting behavioral health disorders. Antidepressants: patients who were prescribed at least one antipsychotic medication at the time of COVID-19 infection. CGI-S: Clinical Global Impression severity scales. T0_CGI:_CGI-S scales from September 1^st^ 2019 to the date of COVID-19 infection (pre-COVID); T1_CGI: CGI-S scales from the date of COVID-19 till October 22^nd^ 2020 (post-COVID). SD: standard deviation. IQR .25: the 25% bound of Interquartile range. IQR .75: the 75% bound of Interquartile range.
(XLSX)

S3 Table Mortality in patients with and without concurrent antipsychotics use. Multivariable logistic regression adjusting for age, gender, antidepressant use, living arrangement and dementia diagnosis.
(XLSX)

S4 Table Patient characteristics stratified by antipsychotics use of COVID-19 patients. Antipsychotics: patients who were prescribed at least one antipsychotic medication at the time of COVID-19 infection to treat preexisting behavioral health disorders. Institutional Living is defined as living at an assisted living facility or at a rehabilitation facility. For the association between antipsychotics and age, logistic regression was used. For the associations of binary variables, Fisher’s exact test was used. P-values are unadjusted for covariates.
(XLSX)

## References

[1] Koponen M, Taipale H, Lavikainen P, Tanskanen A, Tiihonen J, Tolppanen AM, et al. Risk of Mortality Associated with Antipsychotic Monotherapy and Polypharmacy Among Community-Dwelling Persons with Alzheimer’s Disease. J Alzheimers Dis. 2017;56(1):107–18. doi: 10.3233/JAD-160671 . 27935554

[2] Schneeweiss S, Setoguchi S, Brookhart A, Dormuth C, Wang PS. Risk of death associated with the use of conventional versus atypical antipsychotic drugs among elderly patients. CMAJ. 2007;176(5):627–32. doi: 10.1503/cmaj.061250 ; PubMed Central PMCID: PMC1800321. 17325327 PMC1800321

[3] Wang PS, Schneeweiss S, Avorn J, Fischer MA, Mogun H, Solomon DH, et al. Risk of death in elderly users of conventional vs. atypical antipsychotic medications. N Engl J Med. 2005;353(22):2335–41. doi: 10.1056/NEJMoa052827 . 16319382

[4] Ostuzzi G, Papola D, Gastaldon C, Schoretsanitis G, Bertolini F, Amaddeo F, et al. Safety of psychotropic medications in people with COVID-19: evidence review and practical recommendations. BMC Med. 2020;18(1):215. Epub 2020/07/16. doi: 10.1186/s12916-020-01685-9 ; PubMed Central PMCID: PMC7360478. 32664944 PMC7360478

[5] Schneider-Thoma J, Efthimiou O, Bighelli I, Dorries C, Huhn M, Krause M, et al. Second-generation antipsychotic drugs and short-term somatic serious adverse events: a systematic review and meta-analysis. Lancet Psychiatry. 2019;6(9):753–65. Epub 2019/07/20. doi: 10.1016/S2215-0366(19)30223-8 . 31320283

[6] May M, Slitzky M, Rostama B, Barlow D, Houseknecht KL. Antipsychotic-induced immune dysfunction: A consideration for COVID-19 risk. Brain Behav Immun Health. 2020;6:100097. Epub 2020/08/25. doi: 10.1016/j.bbih.2020.100097 ; PubMed Central PMCID: PMC7308744. 32835296 PMC7308744

[7] Yang W, Kandula S, Huynh M, Greene SK, Van Wye G, Li W, et al. Estimating the infection-fatality risk of SARS-CoV-2 in New York City during the spring 2020 pandemic wave: a model-based analysis. Lancet Infect Dis. 2020. Epub 2020/10/23. doi: 10.1016/S1473-3099(20)30769-6 ; PubMed Central PMCID: PMC7572090. 33091374 PMC7572090

[8] Cummings MJ, Baldwin MR, Abrams D, Jacobson SD, Meyer BJ, Balough EM, et al. Epidemiology, clinical course, and outcomes of critically ill adults with COVID-19 in New York City: a prospective cohort study. Lancet. 2020;395(10239):1763–70. Epub 20200519. doi: 10.1016/S0140-6736(20)31189-2 ; PubMed Central PMCID: PMC7237188. 32442528 PMC7237188

[9] Pastor-Barriuso R, Perez-Gomez B, Hernan MA, Perez-Olmeda M, Yotti R, Oteo-Iglesias J, et al. Infection fatality risk for SARS-CoV-2 in community dwelling population of Spain: nationwide seroepidemiological study. BMJ. 2020;371:m4509. Epub 2020/11/29. doi: 10.1136/bmj.m4509 ; PubMed Central PMCID: PMC7690290 at www.icmje.org/coi_disclosure.pdf and declare: support from the Spanish Ministry of Health, Institute of Health Carlos III, and Spanish National Health System for the submitted work; no relationships with any organisations that might have a financial interest in the submitted work in the previous three years; no other relationships or activities that could appear to have influenced the submitted work. 33246972 PMC7690290

[10] Bhopal SS, Bhopal R. Sex differential in COVID-19 mortality varies markedly by age. Lancet. 2020;396(10250):532–3. Epub 2020/08/18. doi: 10.1016/S0140-6736(20)31748-7 ; PubMed Central PMCID: PMC7426105. 32798449 PMC7426105

[11] Harrison SL, Fazio-Eynullayeva E, Lane DA, Underhill P, Lip GYH. Comorbidities associated with mortality in 31,461 adults with COVID-19 in the United States: A federated electronic medical record analysis. PLoS Med. 2020;17(9):e1003321. Epub 2020/09/11. doi: 10.1371/journal.pmed.1003321 ; PubMed Central PMCID: PMC7482833 following competing interests. GYHL is a Consultant for Bayer/Janssen, BMS/Pfizer, Medtronic, Boehringer Ingelheim, Novartis, Verseon, and Daiichi-Sankyo and a Speaker for Bayer, BMS/Pfizer, Medtronic, Boehringer Ingelheim, and Daiichi-Sankyo. No fees are directly received personally. DAL has received investigator-initiated educational grants from Bristol-Myers Squibb (BMS) and Boehringer Ingelheim; has been a speaker for Boehringer Ingelheim and BMS/Pfizer; and has consulted for BMS, Boehringer Ingelheim, and Daiichi-Sankyo. EF-E and PU are employees of TriNetX. SLH has declared no competing interests. 32911500 PMC7482833

[12] Ioannou GN, Locke E, Green P, Berry K, O’Hare AM, Shah JA, et al. Risk Factors for Hospitalization, Mechanical Ventilation, or Death Among 10131 US Veterans With SARS-CoV-2 Infection. JAMA Netw Open. 2020;3(9):e2022310. Epub 2020/09/24. doi: 10.1001/jamanetworkopen.2020.22310 ; PubMed Central PMCID: PMC7512055. 32965502 PMC7512055

[13] Brandén M, Aradhya S, Kolk M, Härkönen J, Drefahl S, Malmberg B, et al. Residential context and COVID-19 mortality among adults aged 70 years and older in Stockholm: a population-based, observational study using individual-level data. Lancet Healthy Longevity. 2020;1: e80–88. Epub October 27, 2020. doi: 10.1016/S2666-7568(20)30016-7 33521770 PMC7832817

[14] Arnold C. Could COVID delirium bring on dementia? Nature. 2020;588(7836):22–4. Epub 2020/12/04. doi: 10.1038/d41586-020-03360-8 . 33268868

[15] Varatharaj A, Thomas N, Ellul MA, Davies NWS, Pollak TA, Tenorio EL, et al. Neurological and neuropsychiatric complications of COVID-19 in 153 patients: a UK-wide surveillance study. Lancet Psychiatry. 2020;7(10):875–82. doi: 10.1016/S2215-0366(20)30287-X ; PubMed Central PMCID: PMC7316461. 32593341 PMC7316461

[16] Ward CF, Figiel GS, McDonald WM. Altered Mental Status as a Novel Initial Clinical Presentation for COVID-19 Infection in the Elderly. Am J Geriatr Psychiatry. 2020;28(8):808–11. Epub 2020/05/20. doi: 10.1016/j.jagp.2020.05.013 ; PubMed Central PMCID: PMC7227566. 32425470 PMC7227566

[17] Taquet M, Luciano S, Geddes JR, Harrison PJ. Bidirectional associations between COVID-19 and psychiatric disorder: retrospective cohort studies of 62 354 COVID-19 cases in the USA. Lancet Psychiatry. 2021;8(2):130–40. doi: 10.1016/S2215-0366(20)30462-4 ; PubMed Central PMCID: PMC7820108. 33181098 PMC7820108

[18] Hossain MM, Tasnim S, Sultana A, Faizah F, Mazumder H, Zou L, et al. Epidemiology of mental health problems in COVID-19: a review. F1000Res. 2020;9:636. Epub 20200623. doi: 10.12688/f1000research.24457.1 ; PubMed Central PMCID: PMC7549174. 33093946 PMC7549174

[19] Hao F, Tan W, Jiang L, Zhang L, Zhao X, Zou Y, et al. Do psychiatric patients experience more psychiatric symptoms during COVID-19 pandemic and lockdown? A case-control study with service and research implications for immunopsychiatry. Brain Behav Immun. 2020;87:100–6. Epub 20200427. doi: 10.1016/j.bbi.2020.04.069 ; PubMed Central PMCID: PMC7184991. 32353518 PMC7184991

[20] Busner J, Targum SD. The clinical global impressions scale: applying a research tool in clinical practice. Psychiatry (Edgmont). 2007;4(7):28–37. ; PubMed Central PMCID: PMC2880930. 20526405 PMC2880930

[21] von Elm E, Altman DG, Egger M, Pocock SJ, Gøtzsche PC, Vandenbroucke JP, et al. The Strengthening the Reporting of Observational Studies in Epidemiology (STROBE) statement: guidelines for reporting observational studies. PLoS Med. 2007;4(10):e296. doi: 10.1371/journal.pmed.0040296 ; PubMed Central PMCID: PMC2020495. 17941714 PMC2020495

[22] R Core Team. R: A language and environment for statistical computing. 2019.

[23] Jewell NP. Statistics for epidemiology. Boca Raton: Chapman & Hall/CRC; 2004. xiv, 333 p. p.

[24] Szumilas M. Explaining odds ratios. J Can Acad Child Adolesc Psychiatry. 2010;19(3):227–9. ; PubMed Central PMCID: PMC2938757. 20842279 PMC2938757

[25] Ho FK, Petermann-Rocha F, Gray SR, Jani BD, Katikireddi SV, Niedzwiedz CL, et al. Is older age associated with COVID-19 mortality in the absence of other risk factors? General population cohort study of 470,034 participants. PLoS One. 2020;15(11):e0241824. Epub 20201105. doi: 10.1371/journal.pone.0241824 ; PubMed Central PMCID: PMC7644030. 33152008 PMC7644030

[26] Li L, Li F, Fortunati F, Krystal JH. Association of a Prior Psychiatric Diagnosis With Mortality Among Hospitalized Patients With Coronavirus Disease 2019 (COVID-19) Infection. JAMA Netw Open. 2020;3(9):e2023282. doi: 10.1001/jamanetworkopen.2020.23282 ; PubMed Central PMCID: PMC7527869. 32997123 PMC7527869

[27] Gordon DE, Hiatt J, Bouhaddou M, Rezelj VV, Ulferts S, Braberg H, et al. Comparative host-coronavirus protein interaction networks reveal pan-viral disease mechanisms. Science. 2020;370(6521). doi: 10.1126/science.abe9403 . 33060197 PMC7808408

[28] Hoertel N, Sanchez-Rico M, Vernet R, Beeker N, Jannot AS, Neuraz A, et al. Association between antidepressant use and reduced risk of intubation or death in hospitalized patients with COVID-19: results from an observational study. Mol Psychiatry. 2021. doi: 10.1038/s41380-021-01021-4 . 33536545

[29] Ferini-Strambi L, Salsone M. COVID-19 and neurological disorders: are neurodegenerative or neuroimmunological diseases more vulnerable? J Neurol. 2020. doi: 10.1007/s00415-020-10070-8 ; PubMed Central PMCID: PMC7372546. 32696341 PMC7372546

[30] Steardo L Jr., Steardo L, Verkhratsky. Psychiatric face of COVID-19. Transl Psychiatry. 2020;10(1):261. doi: 10.1038/s41398-020-00949-5 ; PubMed Central PMCID: PMC7391235. 32732883 PMC7391235

[31] Kompaniyets L, Pennington AF, Goodman AB, Rosenblum HG, Belay B, Ko JY, et al. Underlying Medical Conditions and Severe Illness Among 540,667 Adults Hospitalized With COVID-19, March 2020-March 2021. Prev Chronic Dis. 2021;18:E66. Epub 20210701. doi: 10.5888/pcd18.210123 ; PubMed Central PMCID: PMC8269743. 34197283 PMC8269743

[32] Demeco A, Marotta N, Barletta M, Pino I, Marinaro C, Petraroli A, et al. Rehabilitation of patients post-COVID-19 infection: a literature review. J Int Med Res. 2020;48(8):300060520948382. doi: 10.1177/0300060520948382 ; PubMed Central PMCID: PMC7450453. 32840156 PMC7450453

