# Supplementary material for: Mortality in association with antipsychotic medication use and clinical outcomes among geriatric psychiatry outpatients with COVID-19
Source: PLoS One. 2021 Oct 21;16(10):e0258916. doi: 10.1371/journal.pone.0258916 (PMC8530340; doi:10.1371/journal.pone.0258916)
Supplement: Attachment — Submitted filename: PONE_response_20210923.docx [file pone.0258916.s006.docx]

**Response to editor and reviewers**

*We thank the editor for the suggestion to modify the title of our manuscript.*

Editor Comments:

Thank you for the revision. Can you please modify your title to: “Mortality and clinical outcomes in association with antipsychotic medication use among geriatric psychiatry outpatients with COVID-19"

*> We edited the title. We investigated mortality, not clinical outcome, in association with antipsychotic medication use. We described clinical outcome in our cohort. So we changed the title to “Mortality in association with antipsychotic medication use and clinical outcomes among geriatric psychiatry outpatients with COVID-19”.*
